# Response of the Authors

**DOI:** 10.1111/anec.12852

**Published:** 2021-05-29

**Authors:** Luca Bergamaschi, Pasquale Paolisso, Francesco Angeli, Michele Fabrizio, Andrea Rinaldi, Alberto Foà, Carmine Pizzi

**Affiliations:** ^1^ Unit of Cardiology Department of Experimental, Diagnostic and Specialty Medicine‐DIMES IRCCS. S. Orsola Hospital University of Bologna Bologna Italy

We thank Murat and his colleagues (Murat et al., 2021) for their interest in our article. As highlighted, the main novelty of our paper is the prognostic role of serial ECG findings in a consecutive hospitalized population of patients with SARS‐CoV‐2‐related pneumonia aiming to track the unfavorable course of patients with COVID‐19 (Bergamaschi et al., 2021). We agree that our study had some differences when compared to other series (Angeli et al., 2020).

In particular, we enrolled consecutive a larger population of patients with laboratory‐confirmed SARS‐CoV‐2 infection and radiological findings suggestive of interstitial pneumonia, including those requiring intensive care with a worsen disease and a higher rate of adverse events. We found that among patients with severe COVID‐19, the ECG pathological abnormalities were more significant, as the ECG alterations after 7 days of hospitalization were more frequently associated with higher FiO2 needed (*P*‐value <.001) and lower PaO2/FiO2 ratio (*P*‐value <.001; Bergamaschi et al., 2021).

Our study was focused on the electrocardiographic alterations during the hospitalization of COVID‐19 pneumonia regardless of the infection onset and independently of other factors such as an acute worsening of the disease. To reach such purpose, we have consecutively collected ECGs both at baseline and after 7 days of hospitalization. We believe that from a clinical point of view, this information at the beginning of hospitalization may be more useful to stratify the risk of major events rather than the general onset of infection which, as we know, can be misleading and therefore difficult to evaluate.

We agree that concomitant treatments and potential drug interactions can cause ECG changes. Meriglier et al. evaluated the safety of two antiviral drugs analyzing standard ECG obtained before starting treatment and up to 7 days in a small population of COVID‐19 patients. They stated that 8 patients developed ECG abnormalities during the antiviral treatment, mainly repolarization disorders, and only 3 patients discontinued lopinavir/ritonavir compared with 4 that stopped darunavir/ritonavir due to the ECG alterations (Meriglier et al., 2021). At the beginning of the pandemic, in our hospital only lopinavir/ritonavir was administrated. We did not report these data in our work because these antiretroviral treatments were soon abandoned as standard therapy due to the lack of beneficial effects on the disease (Cao et al., 2020). On the other hand, 77.7% of patients in our population were treated with hydroxychloroquine at 7 days of hospitalization. Concerning the fear of major arrhythmia due to QTc interval prolongation, we report that only basal QTc interval was associated with major adverse events whereas the subsequent QTc measurements did not impact prognosis (Bergamaschi et al., 2021). Nevertheless, our data demonstrated no association between long QTc interval induced by hydroxychloroquine and ventricular arrhythmias remarking the cardiologic safety of this drug (Gasperetti et al., 2020).

After more than one  year of this pandemic disease, the main challenge is still the lack of standardized treatments of COVID‐19 pneumonia. In fact, only one antiviral drug (remdesivir) showed a clear benefit in shortening the time to recovery (Beigel et al., 2020). It appears crucial to treat the systemic inflammatory response and thrombotic complications triggered by SARS‐COV2 infection (Marfella et al., 2020; Paolisso et al., 2020). More trials are certainly needed to adequately assess the cardiac injury induced by this complex infectious disease.

## ETHICAL APPROVAL

Written informed consent was waived by the ethics committee of the designated hospital for patients with emerging infectious diseases.

## CONFLICT OF INTEREST

None.

## AUTHOR CONTRIBUTIONS

LB wrote the first draft of the manuscript; PP, AF, and CP wrote sections of the manuscript. FA, MF, and AR revised the article. All authors contributed to manuscript revision and read and approved the submitted version.

## References

[anec12852-bib-0001] Angeli, F. , Spanevello, A. , De Ponti, R. , Visca, D. , Marazzato, J. , Palmiotto, G. , Feci, D. , Reboldi, G. , Fabbri, L. M. , & Verdecchia, P. (2020). Electrocardiographic features of patients with COVID‐19 pneumonia. European Journal of Internal Medicine, 78, 101–106. 10.1016/j.ejim.2020.06.015 32586646 PMC7305928

[anec12852-bib-0002] Beigel, J. H. , Tomashek, K. M. , Dodd, L. E. , Mehta, A. K. , Zingman, B. S. , Kalil, A. C. , Hohmann, E. , Chu, H. Y. , Luetkemeyer, A. , Kline, S. , Lopez de Castilla, D. , Finberg, R. W. , Dierberg, K. , Tapson, V. , Hsieh, L. , Patterson, T. F. , Paredes, R. , Sweeney, D. A. , Short, W. R. , … Lane, H. C. (2020). Remdesivir for the treatment of Covid‐19 — Final report. New England Journal of Medicine, 383, 1813–1826. 10.1056/NEJMoa2007764 PMC7262788 32445440

[anec12852-bib-0003] Bergamaschi, L. , D'Angelo, E. C. , Paolisso, P. , Toniolo, S. , Fabrizio, M. , Angeli, F. , Donati, F. , Magnani, I. , Rinaldi, A. , Bartoli, L. , Chiti, C. , Biffi, M. , Pizzi, C. , Viale, P. , & Galié, N. (2021). The value of ECG changes in risk stratification of COVID‐19 patients. Annals of Noninvasive Electrocardiology, e12815. 10.1111/anec.12815 33512742 PMC7994985

[anec12852-bib-0004] Cao, B. , Wang, Y. , Wen, D. , Liu, W. , Wang, J. , Fan, G. , Ruan, L. , Song, B. , Cai, Y. , Wei, M. , Li, X. , Xia, J. , Chen, N. , Xiang, J. , Yu, T. , Bai, T. , Xie, X. , Zhang, L. , Li, C. , … Wang, C. (2020). A trial of lopinavir‐ritonavir in adults hospitalized with severe Covid‐19. New England Journal of Medicine, 382, 1787–1799. 10.1056/NEJMoa2001282 PMC7121492 32187464

[anec12852-bib-0005] Gasperetti, A. , Biffi, M. , Duru, F. , Schiavone, M. , Ziacchi, M. , Mitacchione, G. , Lavalle, C. , Saguner, A. , Lanfranchi, A. , Casalini, G. , Tocci, M. , Fabbricatore, D. , Salghetti, F. , Mariani, M. V. , Busana, M. , Bellia, A. , Cogliati, C. B. , Viale, P. , Antinori, S. , … Forleo, G. B. (2020). Arrhythmic safety of hydroxychloroquine in COVID‐19 patients from different clinical settings. Europace, 22, 1855–1863. 10.1093/europace/euaa216 32971536 PMC7543547

[anec12852-bib-0006] Marfella, R. , Paolisso, P. , Sardu, C. , Bergamaschi, L. , D'Angelo, E. C. , Barbieri, M. , Rizzo, M. R. , Messina, V. , Maggi, P. , Coppola, N. , Pizzi, C. , Biffi, M. , Viale, P. , Galié, N. , & Paolisso, G. (2020). Negative impact of hyperglycaemia on tocilizumab therapy in Covid‐19 patients. Diabetes & Metabolism, 46, 403–405. 10.1016/j.diabet.2020.05.005 32447102 PMC7241396

[anec12852-bib-0007] Meriglier, E. , Rivoisy, C. , Hessamfar, M. , Bernard, N. , Aureau, I. , Lapoirie, J. , Contis, A. , Sacher, F. , Sacristan, B. , Lahouati, M. , Pedeboscq, S. , Vandenhende, M.‐A. , Bouchet, S. , & Bonnet, F. (2021). Safety of hydroxychloroquine and darunavir or lopinavir in COVID‐19 infection. Journal of Antimicrobial Chemotherapy, 76, 482–486. 10.1093/jac/dkaa441 PMC7717306 33221868

[anec12852-bib-0009] Murat, S. , Babayigit, E. , & Gorenek, B. (2021). Comments on the value of ECG changes in risk stratification of COVID‐19 patients. Ann Noninvasive Electrocardiol, e12841. 10.1111/anec.12841 33742516 PMC8164154

[anec12852-bib-0008] Paolisso, P. , Bergamaschi, L. , D'Angelo, E. C. , Donati, F. , Giannella, M. , Tedeschi, S. , Pascale, R. , Bartoletti, M. , Tesini, G. , Biffi, M. , Cosmi, B. , Pizzi, C. , Viale, P. , & Galié, N. (2020). Preliminary experience with low molecular weight heparin strategy in COVID‐19 patients. Front Pharmacol, 11, 1124. 10.3389/fphar.2020.01124 32848743 PMC7424043

